# Book Review: The Productive Researcher

**DOI:** 10.3389/fnhum.2017.00544

**Published:** 2017-11-07

**Authors:** Mathieu Hainselin

**Affiliations:** Centre de Recherche en Psychologie: Cognition, Psychisme et Organisations (CRP-CPO), EA 7273, Département de Psychologie, Université de Picardie Jules Verne, Amiens, France

**Keywords:** productivity, goal, management, smart, scientific writing

When I was a young student with some hair, wondering if I should become a clinical neuropsychologist or a researcher, I had hundreds of questions about research. Now I am PI and PhD student advisor, have some funded projects, papers published, a long rejection list of articles or grants and…thousands of questions. Why do I do this job? Why keep running after money while I wanted (and still want) to run after knowledge? Mark Reeds helps us to find answers and gives tips to many of these questions. In fact, he gives answers to questions I was not even asking but wish I asked myself earlier, such as how, when and whom I can say yes or no to. If you never question anything about yourself or science, this book review and The Productive Researcher (Reed, 2017) are not for you. If you are or know some researchers experiencing difficulties to have a satisfactory work/life balance, a very common issue including in biomedical science (Holleman et al., 2015), you will probably enjoy this book. It is divided in two parts: “Principles” and “Making it happen,” with general advices and well-designed quizzes to question yourself and find your own path and key lessons summed up after each chapter. Overall, the book is a good mix between 20% of science foundation, 30% of Mark Reed's personal anecdotes, and 50% of compiled tips from world most productive researchers. The first chapter is a long introduction with some storytelling of the author's personal uneasy history and the problems of techniques supposed to work for everyone.

The second chapter, first of the “Principles” part, leads the reader to focus on our motivations and find which are the most important to us. The approach is based on the Relational Frame Theory (RFT; Hayes, 2004): it proposes that language and cognition are developed by creating links between concepts and objects and linking items to one another. Chapter 3 is about retell your story and gives a helpful tool with an identity pie (what I am) and a time pie (what I spend time on). The gap between the pies helps to identify what activity we might be neglecting. Both chapters 2 and 3 are useful tools to fight against the very common impostor syndrome (feeling you are a fraud, not deserving to be a scientist although you have academic success). The single-mindedly prioritize chapter 4 starts with empathy, humility, and trust, needed to empower our colleagues and ourselves. It is not what you (think you) should do that matters, but focusing on the important unfinished task linked to your core goals. Emails, and how not to let them eat up us, is usually not on the top of the prioritization list evoked here. Chapter 5 introduces the new SMART (Specific, Measurable, Achievable, Realistic, and Timely) goals, inspired by the classic Locke and Latham (2002) 35 years of empirical research on goal-setting article. Mark Reeds insists on setting the SMART goals in group as we need to achieve them in group.

The “Make it happen” part first chapter, sixth in the book, is entitled “plan that work” and dedicated to turn goals into action, with three questions: “what works?”, “what will you do?” and “how will you know it worked?”. From here, all little rocks the author left in the first part help us to find our path within the forest-pictured book. Chapter 8 focus on saying “yes” to say “no.” Because saying no can be difficult for us to say and for other people to hear, one amongst many advices is to say yes to explain why we are saying no. A very informative table with yes and no situations helps scientists to find inspiration and let the Fear Of Missing Out (FOMO) go. It also debunks the myth of waiting uninterrupted time to write. The example of a 1-week written literature review starts with breaking every writing task down into different-sized chunks, a process similar to the efficient and popular neuropsychological Goal Management Training (Levine et al., 2011). Chapter 9 introduces the “do less to do more” motto by forming high-performing writing circle and the need to rest. Chapter 10 title is probably a sufficient reason to read the book: “How to spend less time on emails and meetings.” Setting-up out-of-office for the week emails when we have a deadline and asking ourselves if our presence to the next meeting is absolutely essential are some advices many will find useful, with a specific part for meeting chair and their responsibilities in time-wasting. Chapter 11 deals with time spent online. To stop wasting time and start driving impact on social media, few questions and some dedicated apps can help us to manage our digital footprint.

The conclusive last chapter highlights the power of social norms and our need to have some backup. To help each other in our scientific community, one way is to have as many people as possible to read this book.

Overall, The Productive Researcher will be useful for scientists, not only those working in human neuroscience, but also clinicians. The interviews with some of the world's highest performing researcher, combined with interdisciplinary literature and Mark Reeds' own experience, can help all of us to be more productive, without overworking. In fact, this book will probably help scientists to work less and be happier.

## Author contributions

The author confirms being the sole contributor of this work and approved it for publication.

### Conflict of interest statement

The author declares that the research was conducted in the absence of any commercial or financial relationships that could be construed as a potential conflict of interest.
